# Dental Expositor

**Published:** 1853-10

**Authors:** 


*Dental Expositor.*—The editor of this semi-annual, Dr. Solyman Brown, commenced, in the May number, the republication of his treatise on Mechanical Dentistry, which appeared about twelve years since in this Journal. The author has thoroughly revised it, and will add all the improvements which have been made subsequently, in this department of dental practice. Dr. B. is known to our readers as an accomplished scholar and writer, and of his ability to produce a valuable treatise on mechanical dentistry, there can be no doubt. The Expositor is furnished to subscribers at twenty-five cents a year, and separate copies of the treatise on Mechanical Dentistry, printed on thick vellum paper, bound at the close of the volume in handsome muslin, can be had for one dollar, paid in advance, or two dollars when completed. The value of the work will, we feel assured, commend it to the profession, and more especially to its younger members. We hope the editor may be liberally remunerated for his labor.

